# A semi-independent policies training method with shared representation for heterogeneous multi-agents reinforcement learning

**DOI:** 10.3389/fnins.2023.1201370

**Published:** 2023-06-19

**Authors:** Biao Zhao, Weiqiang Jin, Zhang Chen, Yucheng Guo

**Affiliations:** ^1^School of Information and Communications Engineering, Xi'an Jiaotong University, Xi'an, China; ^2^School of Software Engineering, Xi'an Jiaotong University, Xi'an, China; ^3^Key Laboratory of Shaanxi Province for Craniofacial Precision Medicine Research, College of Stomatology, Xi'an Jiaotong University, Xi'an, China; ^4^Department of Orthodontics, Stomatological Hospital of Xi'an Jiaotong University, Xi'an, China

**Keywords:** brain function, knowledge-sharing institution, multi-agent reinforcement learning, parameters sharing, representation transferability

## Abstract

Humans do not learn everything from the scratch but can connect and associate the upcoming information with the exchanged experience and known knowledge. Such an idea can be extended to cooperated multi-reinforcement learning and has achieved its success on homogeneous agents by means of parameter sharing. However, it is difficult to straightforwardly apply parameter sharing when dealing with heterogeneous agents thanks to their individual forms of input/output and their diverse functions and targets. Neuroscience has provided evidence that our brain creates several levels of experience and knowledge-sharing mechanisms that not only exchange similar experiences but also allow for sharing of abstract concepts to handle unfamiliar situations that others have already encountered. Inspired by such a brain's functions, we propose a semi-independent training policy method that can well tackle the conflict between parameter sharing and specialized training for heterogeneous agents. It employs a shared common representation for both observation and action, enabling the integration of various input and output sources. Additionally, a shared latent space is utilized to maintain a balanced relationship between the upstream policy and downstream functions, benefiting each individual agent's target. From the experiments, it can approve that our proposed method outperforms the current mainstream algorithms, especially when handling heterogeneous agents. Empirically, our proposed method can also be improved as a more general and fundamental heterogeneous agents' reinforcement learning structure for curriculum learning and representation transfer. All our code is open and released on https://gitlab.com/reinforcement/ntype.

## 1. Introduction

The attention on Multi-agent reinforcement learning (MARL) is booming largely since a lot of real-world cooperatives challenges can be properly solved. The scenarios such as distributed network routers, sensor networks (Zhang and Lesser, 2011), traffic management (Singh et al., 2020), and coordination of robot swarms (Hüttenrauch et al., 2017), etc. can be better modeled as MARL where the decision on controlling and management are distributed made. However, the introduction of multi-agent reinforcement learning has also brought in 2 challenges: increased computation requirements due to the larger observation and action spaces, and difficulty in convergence during training due to the presence of other agents.

Multi-Agent Reinforcement Learning (MARL) methods can be classified into two categories based on the level of centralization in decision-making and learning (centralized or decentralized). In decentralized systems, each agent makes decisions and learns on its own, without accessing the observations, actions, or policies of other agents. However, decentralized learning lacks the guarantee of convergence due to the non-stationary caused by other agents. Therefore, most modern MARL research follows the paradigm of Centralized Training and Decentralized Execution (CTDE), where agents have access to other agents' observations during training but execute their own policies separately. Examples of CTDE include MADDPG (Lowe et al., 2017), COMA (Foerster et al., 2018), and QMIX (Rashid et al., 2020).

Based on such a paradigm, the idea of parameter sharing is naturally born following the merging of multi-reinforcement learning. It coheres to the human intuition that knowledge sharing can make better learning and judgment. Humans do not learn everything from scratch but exchange knowledge when learning from experience. This idea was first introduced for classical RL (Tan, 1993) and later extended to cooperative multi-agent reinforcement learning (Chu and Ye, 2017; Gupta et al., 2017). Homogeneous multi-reinforcement learning has achieved success when utilizing parameter sharing. They leverage an identical policy trained with all the trajectories. This method is more efficient compared to training multiple independent policies, as only one policy is employed for both learning and training, reducing the high computational demands, and difficulties in achieving convergence.

The application of parameter sharing to heterogeneous agents is limited in its effectiveness due to the homogenizing effect it has on agents' behavior, particularly at the early stages. Additionally, the shared policy results in a fixed observation and action space size. To address this, some algorithms utilize zero-padding to standardize inputs and outputs, and allow a single policy to serve multiple agents (Gupta et al., 2017; Foerster et al., 2018). These strategies have helped to reduce the obstacles to further extension to hetero agents. It works well for agents with fewer functional and targeting variations or for environments easy to normalize the input and output but not for an abundance diversity of agents. However, this adaptation may not be suitable for all situations, such as when there are different dimensions of inputs and outputs that are not easy to be unified through extra padding of inputs or outputs. The policy for diverse agents also results in slow convergence. Therefore, a more flexible parameter-sharing and policy training strategy is desirable for the real-world application.

Neuroscience has provided evidence that our brain establishes various levels of experience and knowledge-sharing institutions that not only exchange similar experiences but also allow for the exchange of abstract concepts to tackle novel situations that others have already encountered. Inspired by this, we propose a semi-independent training policy method that applies identical policies among the same type of agents and semi-independent parameter-sharing schemes between different types for tackling the conflict between parameter sharing and specialized training for heterogeneous agents. This method also utilizes a common shared representation, generated by supervised learning, to formalize the observations and actions of the agents, allowing it to handle all types of inputs and outputs. An intrinsic reward is also introduced to speed up the environmental exploration. Experimental results demonstrate that our proposed method outperforms the current mainstream algorithms, particularly when dealing with heterogeneous agents. In advance, our proposed method can be considered as a more general and fundamental structure for heterogeneous agent reinforcement learning, incorporating curriculum learning and representation transferring.

This paper is organized as follows. In Section 2, we provides some background on Multi-agent Reinforcement Learning (MARL) and recent advances in Deep Reinforcement Learning (DRL) relevant to the proposal. Section 3 presents the proposal in detail. In Section 4, we will detail the experiments performed and their results. In Section 5, we will review the related work concerning our proposed MARL, including curriculum learning and representation transferring. Lastly, in Section 6, we will summarize the conclusions and suggests future research directions.

### 1.1. Main contribution

This paper presents three main contributions we have made to our proposal.

First, we introduce and adopt a hard-parameter-sharing scheme to MARL in order to balance the conflicting requirements of agents' specialization and network fast convergence. This scheme was originally proposed for multi-task networks, which take a parameter-shared base to process the input and multiple-task terminals to handle different tasks. This structure accounts for specialization among heterogeneous agents while still attempting for the maximum level of experience sharing. Based on our knowledge, there is no other literature currently existing for this approach, and our work is the first made such attempt to introduce the multi-task network parameter-sharing scheme to multi-agent reinforcement learning.

Second, we invent a supervised learning method to generate a general input and output representation shared with all agents. The shared common representation facilitates the formalization of input and output, thus resolving the diversity of heterogeneous agents' input and output issues, and making it easier to incorporate the hard-parameter-sharing scheme. Thanks to this common shared input/output representation, all the agents will be equally treated after the input/output processing regardless of the types of agents. Empirically, We carried out such an approach by simultaneously training with reinforcement learning to ensure that the representation is both accurate and precise. It can be approved such a training schedule can fast generate the representation to facilitate policy training.

Third, we introduce an extra intrinsic reward to encourage more exploration of the environment initially. Unlike traditional intrinsic rewards which are based on a comparison of trajectories, our proposed intrinsic reward is based on the prediction of supervised learning and its input/output representation. Such a tactic can help to stimulate more exploration right away without requiring extra effort and well incorporate the representation generation process.

## 2. Background

### 2.1. Reinforcement learning

Reinforcement Learning (RL) methods attempt to identify an optimal policy (a function that takes an observation and returns an action) that maximizes the expected total reward from an environment. Commonly, such environments are modeled as a Markov Decision Process (MDP) or Partially-Observable Markov Decision Process (POMDP) (Boutilier, 1996). MDPs characterize decision-making as a repetitive process whereby an agent takes an action, receives a reward, and transitions to a new state (with perfect knowledge of the state). POMDP extends this to include environments in which the agent may not be able to observe the full state information.

In Deep Reinforcement Learning (DRL), a neural network is used to represent the policy. These methods are typically divided into two categories: Q-learning methods and policy gradient (PG) methods. The first deep Q learning method was Deep Q Network (DQN) (Mnih et al., 2013), and the first widely-used PG method was Deep Deterministic Policy Gradient (DDPG) (Lillicrap et al., 2015). Subsequently, various newer, more powerful methods were developed, including Soft Actor-Critic (SAC) (Haarnoja et al., 2018), TD3 (Fujimoto et al., 2018), Proximal Policy Optimization (PPO) (Chu and Ye, 2017), (the synchronous version of Asynchronous Advantage Actor-Critic (A3C) (Mnih et al., 2016), Rainbow DQN (Hessel et al., 2018) etc., and more advanced deep reinforcement learning methods is on the way of development for the real-world applications.

Multi-agent reinforcement learning (MARL) can be deemed as an extension of RL that considers the interactions between multiple agents in a changing environment. The agents must learn to adjust their actions based on changes not only in the environment but also in the behavior of other agents. MARL can lead to distributed intelligent decision-making and has applications in game theory and robotics. Our proposed method focuses on developing a fast and accurate MARL algorithm for practical use.

### 2.2. Brain's transfer learning on the new tasks

Learning is not a process that begins from scratch, as people can connect and relate new information to their existing experiences and knowledge. Recent neuroscience research has shown that the brain has the capacity to transfer knowledge from one task to another, even if the tasks appear dissimilar. The brain's ability to extract and store abstract representations of information is the reason behind this transfer. When confronted with a new task, the brain first looks for similarities with past experiences, allowing individuals to learn how to handle the new task quickly. These abstract experiences can also be shared and learned by others, highlighting the importance of utilizing past experiences and knowledge to facilitate learning.

### 2.3. Dec-POMDP

Decentralized Partially Observable Markov Decision Processes (Dec-POMDPs) are a probabilistic framework for enabling distributed decision-making among multiple agents. It has been commonly utilized for decision-making in cooperated large-scale multi-agent settings, originally proposed in the literature on autonomous multi-agent systems (Lillicrap et al., 2015). In this framework, each agent has a set of actions and observations defined in mathematics that it can take in order to achieve a goal. The environment is represented as a stochastic process that is partially observable to the agents.

A Dec-POMDP on MARL can be formally defined as a tuple (*N*, S, *A, P, R*, Ω, O, n, γ), where:

- *N* is a finite state of n agents where *i*∈*N*≡{1, …, *n*};
- *S* is the global state of the environment where *s*∈*S*;
- *A* is a set of joint actions, $A=A_{1}\times \cdots \times A_{N}$ where $A_{i}$ is the set of actions that the *i*-th agent can choose from;
- *P* is a state transition probability function where $P\left(s^{{}^{\prime }}|s,A\right):\text{S}\times \prod_{i\in N}A_{i}\times \text{S}\to \left[0,1\right]$;
- *R* is a reward function, often can be modeled as *R* = R(S, *A*), where $R_{i}\in R:\text{S}\times \prod_{i\in N}A_{i}\times \text{S}\to \mathbb{R}$ is the reward function for agent *i*;
- Ω is the set of observations, where Ω_*i*_∈Ω is the possible observation for agent *i*;
- *O* is the observation function, normally modeled as *O*(*S, i*). According to the settings of partial observation, the agent cannot access the global state but samples local observations according to the observation function where $S\times A_{i}\equiv \Omega_{i}$, which can generate the set of observation that *i*th agent can receive;
- γ is the discount factor, where γ∈[0, 1). The utilization of the discount factor is to compromise for the reward one can receive a few steps later than immediately.

The set of agents *A* comprises the agents that are involved in the decision-making problem, each of which has its own set of decisions and observations. The set of observed states *S* represents the states of the environment, which are partially observed by the agents. Finally, the set of joint actions *A* contains the joint actions taken by all, which are finally to determine the probability of transitioning to different states. The Dec-POMDP framework allows agents to make optimal decisions in a partially observable environment by combining their observations and taking into account their own rewards and the rewards of their peers (Oliehoek, 2012).

Such a framework can be perfectly utilized to describe the decision-making in cooperated large-scale multi-agent settings, thus we will also apply the above-mentioned mathematics definitions in this paper to describe our proposal.

### 2.4. Parameters sharing

The concept of parameter sharing is a widespread practice in the field of deep learning. It refers to an approach where a single set of parameters is shared among multiple components of a neural network, such as layers or sub-networks. In the context of multi-agent reinforcement learning, parameter sharing involves an algorithm that learns from the experiences of all agents and updates a collectively shared policy. Parameter sharing, which involves representing all policies with a single neural network that shares the same set of parameters, was first introduced by Tan (1993) for classical reinforcement learning. Later, it was concurrently introduced to cooperative multi-agent deep reinforcement learning by Chu and Ye (2017) and Gupta et al. (2017). This straightforward approach has proven to be highly effective in various applications, including those presented in Zheng et al. (2018), Chen et al. (2021), and Yu et al. (2022). This paper will discuss parameter sharing in detail and make a proposal based on that with a more general framework and structure from the common representation and semi-independent training and will further analyze the effectiveness and utilization of representation transferring and curriculum learning.

### 2.5. Coping with heterogeneity

Heterogeneity in agents is a common challenge in multi-agent systems, which can arise due to various reasons, such as differences in the physical capabilities or perceptual abilities of the agents. Addressing this issue is crucial to ensure that the agents can effectively cooperate and achieve their goals. To address such a challenge, two methods have been proposed. The first method is to add an indication of observations to enable a single policy to serve multiple agents, accommodating different action and observation spaces. However, since there is only one neural network, the observation spaces of all agents must be the same size especially when the observation spaces of agents are vastly different, as the neural network may struggle to learn from a disparate input. The second method proposes “padding” observations and action spaces to a uniform size, which allows agents to ignore any actions outside their “true” action space. By standardizing the observation and action spaces, the agents can effectively communicate with each other, and the neural network can learn from these inputs more efficiently. However, this approach may introduce redundant or irrelevant information, leading to additional computational overhead. Also the initial policies it generated with the unified neutral network will be also less efficient and mislead to sub-optimal when the network cannot well recognize the correct information and “padding”.

## 3. Preliminary

### 3.1. Representation learning

Reinforcement learning (RL) involves training an agent through interactions with an environment. This formalism is powerful in its generality, but poses an open-ended problem: how can we design agents that learn efficiently and generalize well, given only sensory information and a scalar reward signal? One solution that is becoming increasingly popular is introducing self-supervised learning. Applying self-supervised learning in RL can help solve problems with high-dimensional state-action spaces and improve sample efficiency by incorporating inductive biases, such as structural information about tasks anden vs, into the representations for better performance.

The UNREAL agent (Jaderberg et al., 2016) introduced unsupervised auxiliary tasks to deep RL, including the Pixel Control task, a Q-learning method that requires predictions of screen changes in discrete control environments, which has become a standard in DMLab (Hessel et al., 2019). CPC (Oord et al., 2018) applied contrastive losses over multiple time steps as an auxiliary task for the convolutional and recurrent layers of RL agents, and it has been extended with future action-conditioning (Guo et al., 2018). Recently, PBL (Guo et al., 2020) surpassed these methods with an auxiliary loss of forward and backward predictions in the recurrent latent space using partial agent histories. A small number of model-free methods have attempted to decouple encoder training from the RL loss as ablations, but have met reduced performance relative to end-to-end RL (Laskin et al., 2020). Examples of works that pre-train encoder features in advance using image reconstruction losses, such as the VAE (Kingma and Welling, 2013), PR2 (Finn et al., 2016), and World models (Ha and Schmidhuber, 2018). Other works (Devin et al., 2018; Kipf et al., 2019), apply pre-trained object-centric representations that learn a forward model through contrasting losses. CFM (Yan et al., 2021) introduced a similar technique to learn encoders that support the manipulation of deformable objects through traditional control methods. In this paper, we will leverage an encoder-decoder framework to formalize the various inputs and output for heterogeneous agents.

### 3.2. Hard/soft parameter sharing

**Figure 1:**
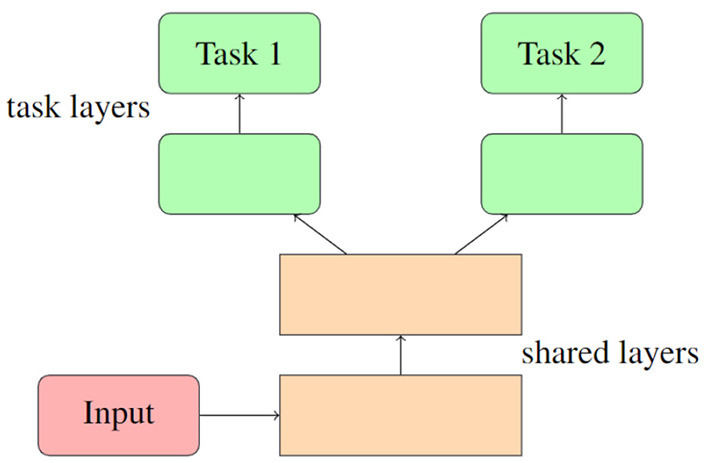
A typical structure of hard parameter sharing.

Hard parameter sharing is a fundamental scheme that enables domains to share some of their model parameters to reduce storage costs and improve prediction accuracy. This approach originated from multi-task learning (MTL), which aims to support multiple downstream tasks on devices. While recent advancements in model compression have made deploying a single model easier, supporting multiple models on devices remains challenging due to increased bandwidth, energy, and storage costs. To address this challenge, the hard/soft parameter-sharing approach has been employed. Unlike soft parameter sharing, where each task keeps its own model and parameters, hard parameter sharing allows multiple tasks to share some of the model parameters. As depicted in Figure 1, this sharing is commonly applied by sharing the bottom layers among all tasks while keeping several top layers and an output layer task-specific (Ruder, 2017). Hard parameter sharing is often used in designing multi-task deep neural network models (Long et al., 2017; Ruder et al., 2019).

Given its effectiveness in MTL, we believe that utilizing hard parameters can also be a viable solution for sharing policies among different types of agents to share the basis while maintaining dependence.

### 3.3. Role-based learning method

Roles are a fundamental aspect of natural systems, such as ants, bees, and humans, where they are closely related to the division of labor and crucial for labor efficiency. This concept has inspired multi-agent system designers to reduce design complexity by assigning agents with the same roles to specific sub-tasks. However, in such systems, roles and their associated responsibilities are typically predefined using prior knowledge, limiting their generalizability and requiring prior knowledge that may not always be available. To overcome this challenge, Wilson et al. (2010) utilized Bayesian inference to learn a set of roles, while ROMA (Wang et al., 2019) developed a specialization objective to encourage the emergence of roles, method RODE (Wang et al., 2020b) proposes a scalable role-based multi-agent learning method that effectively discovers roles by decomposing the joint action space according to action effects, thereby access to the producing of role selectors and learning of role policies in the reduced spaces. These methods suffer from a limitation in searching for the optimal task decomposition in the full state-action space, resulting in inefficient learning in hard-exploration tasks. Our work is inspired by the concept of role-based policy training, and we propose a method that groups agents by their unit types. Within each group, we implement a full parameter-sharing scheme, while across different groups, we use a semi-sharing parameter scheme. This approach can facilitate faster convergence for agents with similar roles or types while allowing for greater flexibility in learning different strategies or behaviors for agents with different roles or types.

## 4. Proposal

**Figure 2:**
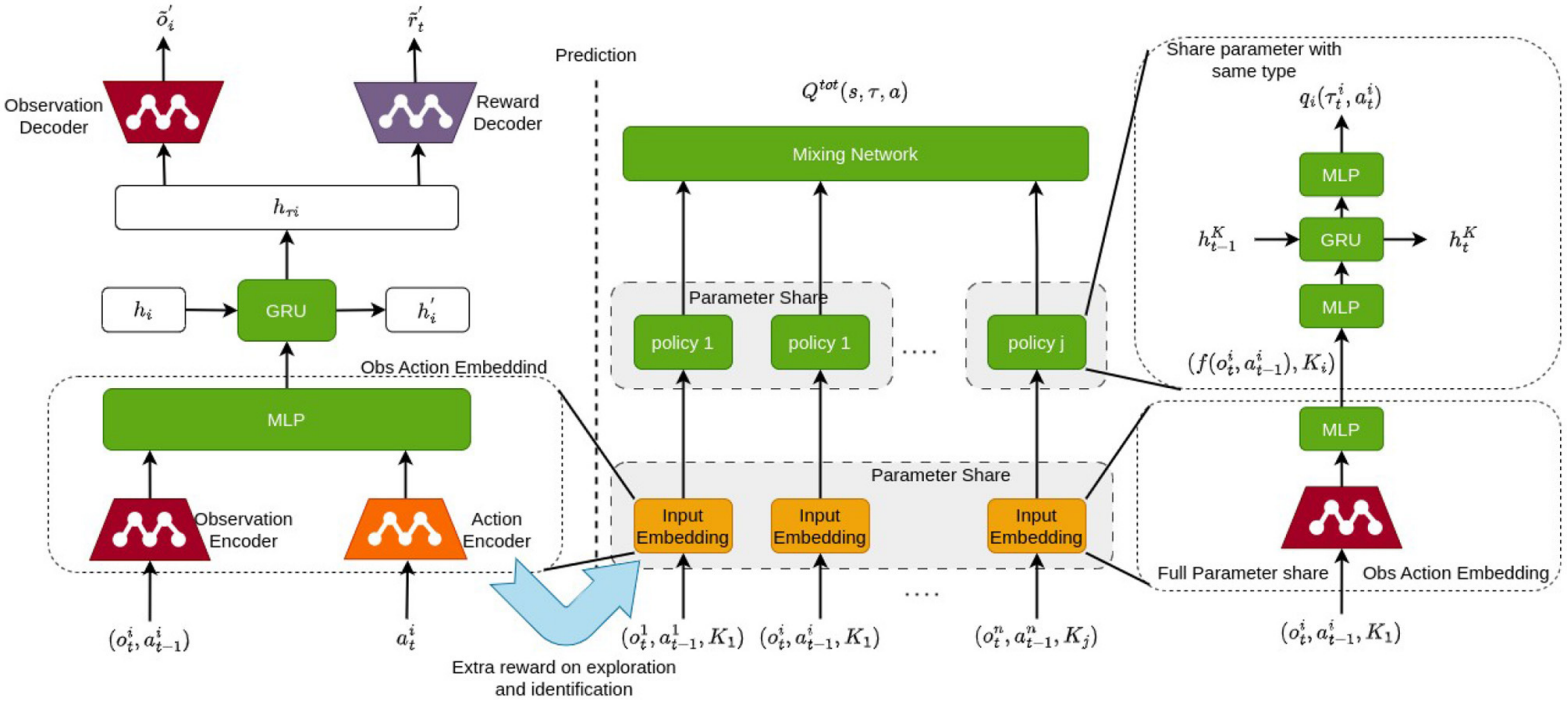
The framework of STSR includes a common representation derived from supervised learning, a semi-independent policy training scheme that applies full shared parameters among agents of the same type and hard share among different types, and an intrinsic/diversity-driven extra reward to encourage environment exploration and enhance the representation that can more clearly distinguish the inputs and outputs of different types.

Based on the preliminary research mentioned above, we propose our semi-independent training policy method with shared representation (STSR) for reinforcement learning. This method comprises three main components: a common inputs/outputs representation derived from supervised learning, a semi-independent policy training scheme that applies full shared parameters among agents of the same type/role and hard sharing among different types, and an intrinsic/diversity-driven extra reward to encourage environment exploration and enhance the representation that can more clearly distinguish the inputs and outputs from different types/roles. Before we delve into each component, Figure 2 depicts the graph illustrating the entire process.

Our idea is to use supervised learning to build a prediction model, which enables us to establish an observation-action embedding to formalize the agent's input and output, regardless of their invariant observation and actions. Based on that, we can extend a hard parameter-sharing scheme to multiple heterogeneous agents, which fully shares the parameters among the same types of agents and employs hard sharing between different type groups. From the learned representation, we will generate an extra intrinsic reward to encourage environment exploration and an identifying reward to enhance the representation difference between agent types. We provide a clear definition of the agent types and representations below.

**Definition 1** *Given a cooperative multi-agent task* *G = (N*, S, *A, P, R*, Ω, O, n, γ)*, let* K_*j*_ *be a set of agent type with the total type accounts for* j*, where each agent* *i*∈K_*j*_*. Each type with the same policy forms as the tuple* (*g*_*j*_, π_*K*_*j*__)*, where* $g_{j}=\left(N_{j},\text{S},A_{j},{\text{P}}_{j},R,\Omega_{j},\text{O},{\text{n}}_{j},\gamma ,{\text{Z}}_{\text{j}}^{\text{o}},{\text{Z}}_{\text{j}}^{\text{a}}\right)$ *can be defined as a sub-space for each type*, π_*K*_*j*__:*T*×*A*_*j*_ → [0, 1] *is a full parameter shared type policy, associated with each type*. ${\text{Z}}_{\text{j}}^{\text{o}}={\text{Z}}^{0}\left(o_{i},K_{j}\right),{\text{Z}}_{\text{j}}^{\text{a}}={\text{Z}}^{a}\left(a_{i},K_{j}\right)$ *are the observation representation function and action representation function, respectively, shared for each type*.

Our aim is to seek a set of hard parameters shared policies π_*K*_*j* that can maximize the expected global return $Q(s_{t},a_{t})=E_{s_{t+1:\infty },a_{t+1:\infty }}[\sum_{i=0}^{\infty }\gamma^{i}r_{t+1}|s_{t},a_{t},K(Z^{o},Z^{a})]$. The policies π_*K*_*j* are also related to each other in terms of basic representation Z^*a*^, Z^*o*^, and low-level layers. We will now introduce the comprised each component in detail, which is illustrated in Figure 2.

### 4.1. Common observation and action representation

To well handle the heterogeneous agents and to improve the effectiveness of parameter sharing, we attempt to cluster the agents according to their types and then exert full parameter sharing among unit type. Even though some role-based MARL (Wang et al., 2020b; Christianos et al., 2021) do the partition of the agents according to their representation latent space, we group our agents based on the agent's unit type.

To formalize the input and outputs from different types of agents and to better architecture the hard parameter sharing schemes, we propose a recurrent neural network (RNN) based prediction model for learning the observation and action latent representation that incentivizes including enough information such that the next observations and rewards can be predicted when given the actions and current observations.

**Figure 3:**
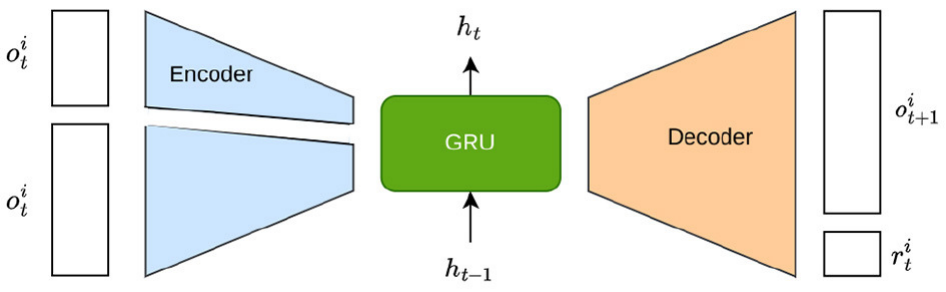
RNN-based prediction model from which to learn the observation and action representation embedding.

As it is depicted in Figure 3, a collection of functions ${\text{Z}}^{0}\left(o_{i},K_{j},t\right)$ and ${\text{Z}}^{a}\left(a_{i},K_{j},t\right)$ are employed to estimate $o_{t+1}^{i}$ and $r_{t+1}^{i}$, respectively, from the agents' limited view of the world. Due to the fact that an agent cannot perceive the state or actions of another agent, we define Ô^*i*^:*O*^*i*^×*A*^*i*^ → Δ(*O*^*i*^) and ${\hat{R}}^{i}:O^{i}\times A^{i}\to \mathbb{R}$ to model the next observation and reward, respectively, based solely on the action and observation of an agent *i*. Our purpose in learning these functions is to ensure a wide acceptable input/output approximation and to establish an initial full share basis for a hard parameter sharing scheme for all the agents regardless of their types. Such prediction model training is due to be processed before the reinforcement learning while the full-parameter shared basis will be kept updated throughout the whole training process of reinforcement learning.

In our proposal, we introduce an encoder *f*_*e*_ and a decoder *f*_*p*_, both parameterized by θ and depicted in Figure 3. The encoder is solely conditioned on the agent's identity. On the other hand, the decoder is split into an observation decoder, $f_{k_{j}}^{o}$, and a reward decoder, $f_{k_{j}}^{r}$, which receives the observation, action, and sampled encoding *z* of agent *i* and try to predict the next observation and reward. Unlike conventional autoencoders, $o_{t}^{i}$ and $a_{t}^{i}$ bypass the encoder and are only received by the decoder. As a result, due to the bottleneck, *z* can encode information only about the agent, such as its reward function ${\hat{R}}^{i}$ or observation transition model ${\hat{O}}^{i}$.

To formalize the process, we assume that each agent's type denoted as *k*_*j*_, represents its observation transition distribution and reward function. We also assume that both the agent's identity and its observation transition distribution can be projected in a latent space, *z*, through the posteriors *q*(*z*|*k*_*j*_) and *p*(*z*|tr = (*o*_*t*+1_, *o*_*t*_, *r*_*t*_, *a*_*t*_)). The objective is to find the posterior *q*(*z*|*k*_*j*_).

The encoder-decoder model is trained with samples from all agents to learn from the experience of all agents, and it will represent the collection of the agent-centered transition and reward functions ${\hat{P}}^{i}$ and ${\hat{R}}^{i}$ for all i∈N. Given the inputs of the decoder, the information of the agent type can only pass through the sample *z*.

This model can be interpreted as a forward model, which is trained by minimizing the following loss function:


(1) $$\begin{array}{l} {ℒ}_{e}\left(\theta_{e}\right)=E_{\left(o,a,r,o^{\prime }\right)\sim D}[\sum_{i}{\left\|f_{o}\left(z_{a_{i}},o_{i},a_{-i}\right)-o_{i}^{\prime }\right\|}_{2}^{2}\\ \;+\lambda_{e}\sum_{i}{\left(f_{r}\left(z_{a_{i}},o_{i},a_{-i}\right)-r\right)}^{2}] \end{array}$$

where *f*_*o*_ and *f*_*r*_ are predictors for observations and rewards, respectively, and parameterized by θ_*e*_. λ_*e*_ is a scaling factor, D is a replay buffer, and the sum is carried out over all agents.

Minimizing the model loss can be done prior to reinforcement learning. We sample actions *a*^*i*^~*A*^*i*^ and store the observed trajectories in a shared experience replay with all agents. We have empirically observed that the data required for this procedure is orders of magnitude less than what is usually required for reinforcement learning, and it can even be reused for training the policies, thus not adding to the sample complexity.

### 4.2. Intrinsic rewards for environment exploration and unit type identification

Multi-agent Reinforcement Learning (MARL) is an effective method for addressing complex decision-making challenges involving multiple agents, where external rewards are present. This approach enables agents to interact with the environment to make optimal decisions, motivated by rewards. A significant challenge for those designing agents is defining a suitable reward function for sequential decision-making tasks in Reinforcement Learning (RL). Additional potential-based rewards, besides extrinsic rewards, do not alter the order of agent behaviors. However, the choice of potential-based or policy-based reward function used to transform the original reward function can impact the sample and computational complexity of RL agents learning from experience in their environment. While this does not change the optimal policy, it can influence the learning process for better or worse.

The aforementioned representation can facilitate the designing of intrinsic rewards on 2 aspects: novelty rewards which encourage the agent to take extra effort on efficient environmental exploration and representability for diversity which can help to form representation more widely identify a different kind of agent.

One of the main challenges in RL is the trade-off between exploitation and exploration: agents must exploit the actions that they know lead to high rewards, but they must also explore new actions and states in order to discover new strategies that may lead to even higher rewards. The data distance between the forward prediction model can provide an additional source of motivation for exploration, beyond the extrinsic rewards provided by the environment.

For simplicity, we can define the state of the environment by combining the observation and rewards of all agents, which can be expressed as $s_{t}=\left\{\left(o_{t}^{i},r_{t}^{i}\right),i\in N\right\},t=0\ldots \infty$. Let *d*(*r*_1_, *r*_2_) be a distance metric between two representation vectors $r_{1},r_{2}\in {\mathbb{R}}^{d}$. One common distance metric is the Euclidean distance.


(2) $$\begin{array}{l} \begin{array}{l} r_{t}^{e}=\underset{i}{\sum^{\text{​}}}p_{i}^{m}||f_{o}(z_{a_{i}},o_{t}^{i},a_{t}^{-i})-o_{t}^{i\prime }|{|}_{2}^{2} \end{array}\\ \;\begin{array}{l} +\lambda_{e}\underset{i}{\sum^{\text{​}}}p_{i}^{m}{\left(f_{r}(z_{a_{i}},o_{t}^{i},a_{t}^{-i})-r_{t}^{i}\right)}^{2} \end{array} \end{array}$$

where $p_{i}^{m}$ is weight when calculating *Q*_*tot*_ that we can obtain from mixer layer. The reward function 2 assigns a positive reward when the current state *s*_*t*_ is situated in a low-density region of the representation space. This low-density region indicates that the state is unique and hasn't been encountered by the agent before. The value of the reward is modified based on the discrepancy between the density estimate determined by the mixer function, denoted as *p*_*m*_, and the overall density estimate. This normalization procedure guarantees that the reward stays within acceptable limits and does not become unreasonably high. As a result, we can determine the intrinsic reward of promoting environmental exploration. It is worth noting that the emphasis on exploration will decrease once the environment has been thoroughly explored. Therefore, we will introduce a discount factor that will gradually decrease during the training process.

We have incorporated an additional intrinsic reward to our design which aims to promote diversity in the representation of the agent's type. One of our key concepts is to implement a specialization policy for agents of the same type. To encourage this behavior, we implement an additional intrinsic reward system that incentives the agent to have similar representations for the same type when having the same kind of inputs and different representations for different types. In order to create a reliable representation-intrinsic reward, we utilize a method that involves calculating the average representation of agents that are of the same type when they receive a positive input. Conversely, we calculate the average representation of the different types of agents to serve as the negative input. By subtracting the negative reward from the positive reward, we obtain a final representation reward. This representation reward can be expressed in the following form:


(3) $$\begin{array}{l} r_{t}^{d}=\sum_{i}p_{i}^{m}[\frac{1}{N_{i}}({\left\|f_{o}\left(z_{a_{i}},o_{t}^{i},a_{t}^{-i}\right)-o_{t}^{i\prime }\right\|}_{2}^{2}\\ \;+\lambda_{e}{\left(f_{r}\left(z_{a_{i}},o_{t}^{i},a_{t}^{-i}\right)-r_{t}^{i}\right)}^{2})\\ \;-\frac{\lambda_{h}}{N_{j}}({\left\|f_{o}\left(z_{a_{j}},o_{t}^{i},a_{t}^{-i}\right)-o_{t}^{i\prime }\right\|}_{2}^{2}\\ \;+\lambda_{e}{\left(f_{r}\left(z_{a_{j}},o_{t}^{i},a_{t}^{-i}\right)-r_{t}^{i}\right)}^{2})],t=0\ldots \infty \end{array}$$

Our total reward after accounting for both these 2 intrinsic rewards is:


(4) $$\begin{array}{l} r_{t}^{tot}=r_{t}+\lambda_{e}r_{t}^{e}+\lambda_{d}r_{t}^{d},t=0\ldots \infty \end{array}$$

**Figure 4:**
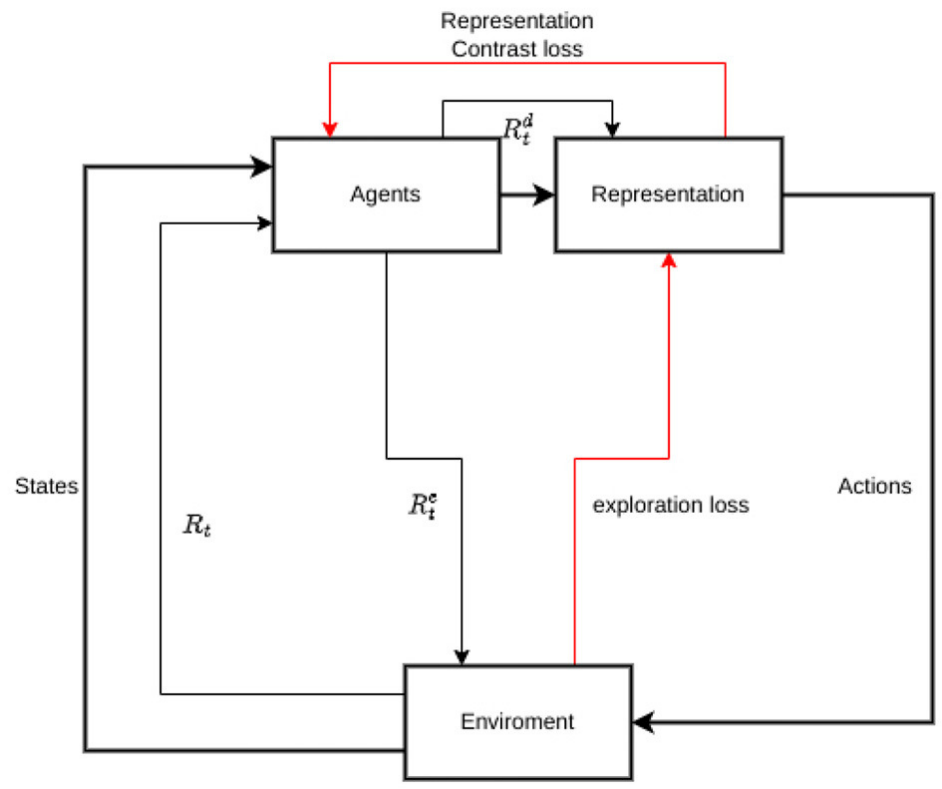
Total rewards including both intrinsic rewards to encourage environment exploration and to diverse representation upon agents' type. Thick black lines illustrate data flow, thin black lines illustrate rewards and red lines illustrate the loss to generate the intrinsic rewards.

This representation, which is demonstrated in Figure 4, incentives intrinsic reward will be taken throughout the whole process of training accompanying the building up with the policy common representation basis.

### 4.3. Common representation based semi-independent policy training

The approach of full parameter sharing has shown remarkable achievements among homogeneous agents. Nevertheless, when extending it to a heterogeneous multi-agent environment, challenges arise regarding how to handle different types of agents with the same policy network that shares all parameters. This extension creates a dilemma since sharing parameters among agents with different characteristics can limit their potential and hinder policy optimization. On the other hand, avoiding parameter sharing altogether requires creating a complex decision-making system with multiple policy networks, each with isolated parameters. This alternative approach leads to slow convergence and inefficient use of experience.

In order to effectively address this issue, we propose utilizing full parameter sharing among agents of the same type, while applying semi-parameter sharing to agents of different types. Agents of the same type share inherent similarities, which enables them to be scaled up with a consistent range of decision-making capabilities. The success of parameter sharing among homogeneous agents supports its application among agents of the same type in a heterogeneous agent system, where the group of heterogeneous agents can be viewed as a collection of multiple sub-groups of homogeneous agents with varying types.

To well utilize the similarities between different sub-groups, we propose to apply hard parameter-sharing schemes. Hard parameter sharing is a technique used in multi-task learning, where a single neural network is trained to perform multiple tasks simultaneously by sharing some of its layers among the tasks. This approach can be effective and efficient because it allows the network to learn and generalize across multiple related tasks, while also reducing the total number of parameters needed to train the model.

Mathematically, hard parameter sharing can be represented as follows: Let *x* be the input to the network, *y*_1_ and *y*_2_ be the outputs of two related tasks, and *f* be the shared layers of the network. Then, the network can be represented as: *y*_1_ = *g*_1_(*f*(*x*)) and *y*_2_ = *g*_2_(*f*(*x*)) where *g*_1_ and *g*_2_ are task-specific output layers. In this way, the shared layers are trained to extract relevant features from the input that are useful for both tasks, while the task-specific output layers are trained to map these features to the desired outputs for each task. By sharing the parameters of the network across tasks, the model can learn to generalize better and improve performance on all tasks.

In the context of multi-agent reinforcement learning, hard parameter sharing can also be useful when different agents share common tasks or goals. For example, in a multi-agent scenario where agents must cooperate to achieve a common objective, such as in a game or robotics application, the agents may share some common knowledge or features that can be learned through a shared network. In our framework, multi-agent reinforcement learning with hard-parameter sharing can be expressed as:


(5) $$\underset{{}_{a_{i}}}{\max}Q(s,a_{i})=\underset{{}_{a_{i}}}{\max}\sum_{t=0}^{\infty }\sum_{i=1}^{n}E_{\pi_{j}}Q_{i}(f(s_{t},a_{i});\theta_{j})$$

*f* denotes the shared layers employed for hard-parameter sharing, while π_*j* = 1…*k*_ represents the policies employed for all agents, where agents of the same type apply the identical policy with full parameter shared.

**Figure 5:**
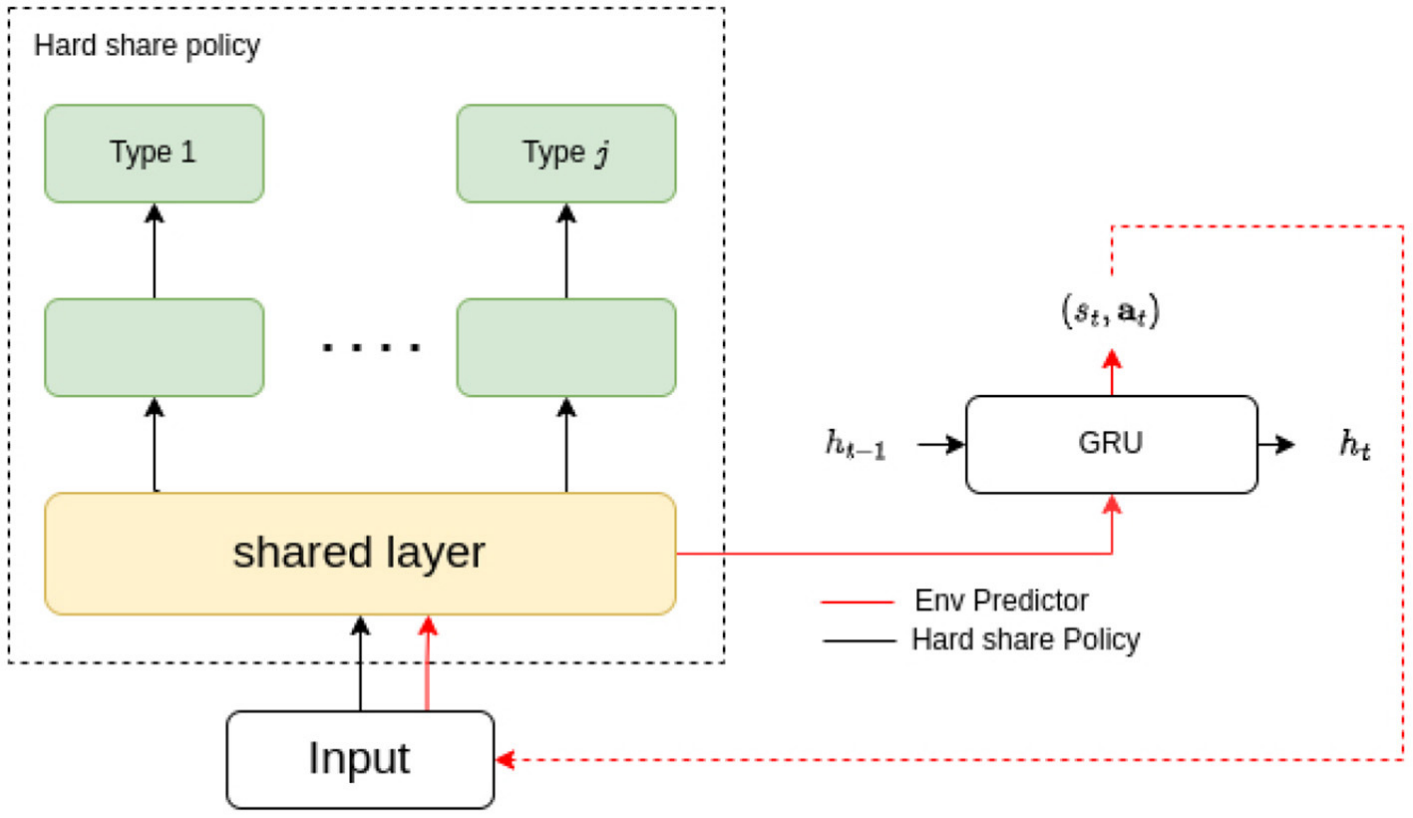
The flow and structure for the hard-parameter-sharing based policy generation scheme, where the initial parameter is from the supervised env prediction and will be later updated along with the type/agent layer.

As illustrated in Figure 5, our proposed shared layer embedding is identical to the common representation latent. The representation latent handles all agent inputs and outputs regardless of unit type, reflecting its parameter-sharing is applicable among all agents. In this case, the representation latent can be selected as the shared layer, initialized with its current parameters. Empirically, this shared layer can be deemed as a separate branch of the common representation, training via unit type based reinforcement learning with the purpose to maximize overall value. In the experiments section, we can prove the shared layer updated with the reinforcement learning outperforms the one updated with the representation latent. Meanwhile, the representation latent is under training with the environment predictor for better representation.

## 5. Experiment and results

In this section, we thoroughly evaluate our proposed method from various perspectives. Firstly, we provide a comprehensive assessment of its overall performance in different scenarios and compare it with other mainstream algorithms to gauge its effectiveness. Secondly, we conduct experiments with different alternative flows and perform ablation studies to assess the impact of each component. Thirdly, we conduct a detailed analysis of the intermediate results to gain a better understanding of the underlying principles, including the initial representation and its subsequent updates, their distribution and representativeness, shared layers, and the course of its training. Finally, we attempt to validate the framework's generalizability by testing its representation transferability and its curriculum learning capacity.

### 5.1. Experiment setting

We have chosen the StarCraft II micromanagement (SMAC) benchmark (Samvelyan et al., 2019) as our test-bed due to its rich environments and high complexity of control. The SMAC benchmark presents a series of challenging tasks, as agents must learn policies in a large action space that includes four cardinal directions, stop, take noop, or select an enemy to attack at each time step. If there are *n*_*e*_ enemies in the map, each ally unit's action space contains *n*_*e*_+6 discrete actions. SMAC environment is rich in all kinds of settings including a lot of homogeneous agents. It is also a widely used setting where multiple agents of distinct types coexist and must learn together, for which our proposed method is mainly focused. The MMM2 is an example of such an environment that contains three types of units (marines, marauders, and medivacs) with distinct attributes. One of the unit types medivacs is particularly different, as it needs to learn how to heal friendly units instead of attacking enemies.

Although our proposal is mainly concerned with heterogeneous agents, it is quite capable to handle all kinds of environments. To conduct a full assessment of our proposal, we carry out tests on all kinds of settings, respectively, regardless of either homogeneous agents or heterogeneous agents settings and we compare the improvements in different settings.

SMAC consists of various maps which have been classified as *easy, hard*, and *super hard*. It also contains variate group agents of homogeneous or heterogeneous. Even though our main proposal is aimed at heterogeneous scenarios, the method is also applicable to the homogeneous and can also outperform its original method.

To fully evaluate its overall performance on different scenarios, we have conducted a thorough evaluation of our approach by benchmarking it across all 14 scenarios within the SMAC suite. This allows us to assess its performance across a range of settings. Additionally, we present some of the results obtained from this evaluation. Furthermore, we have compared our proposal with other value-based MARL algorithms that are considered state-of-the-art, including VDN (Sunehag et al., 2018), QMIX (Rashid et al., 2020), QPLEX (Wang et al., 2021), some role-based MARL method including ROMA (Wang et al., 2020a), and RODE (Wang et al., 2020b) and an agent-specific modules based parameter-sharing algorithm CDS (Li et al., 2021).

To better understand the contribution of each component, we conducted an ablation study by comparing the performance with and without various components. This series of tests were assigned different names: *STSR full* denotes the setting where all components were included, *STSR No Representation Learning* excluded the common representation as the basis for hard-parameter sharing, instead using a random basis initially. The *STSR No Representation Later-update* setting did not update or learn the hard-parameter sharing basis but only utilized the initial common representation. Additionally, we examined the settings of *STSR No* *r*_*e*_ *Reward* and *STSR No* *r*_*d*_ *Reward*, which, respectively, excluded the exploration reward and representation reward. Finally, the *STSR No Hard-Parameter-Share* setting did not apply the hard parameter sharing scheme and did not share parameters among different types of agents.

In the next section, we will present and discuss the results of these thorough evaluation and ablation tests.

### 5.2. Results and discussion

#### 5.2.1. Overall performance

To assess the performance of the models or algorithms, the experiments in this section were conducted 4 times using different random seeds. The median performance is reported as performance metrics. These metrics provide a comprehensive understanding of the models or algorithms' performance and account for the variability that can occur due to stochasticity.

**Figure 6:**
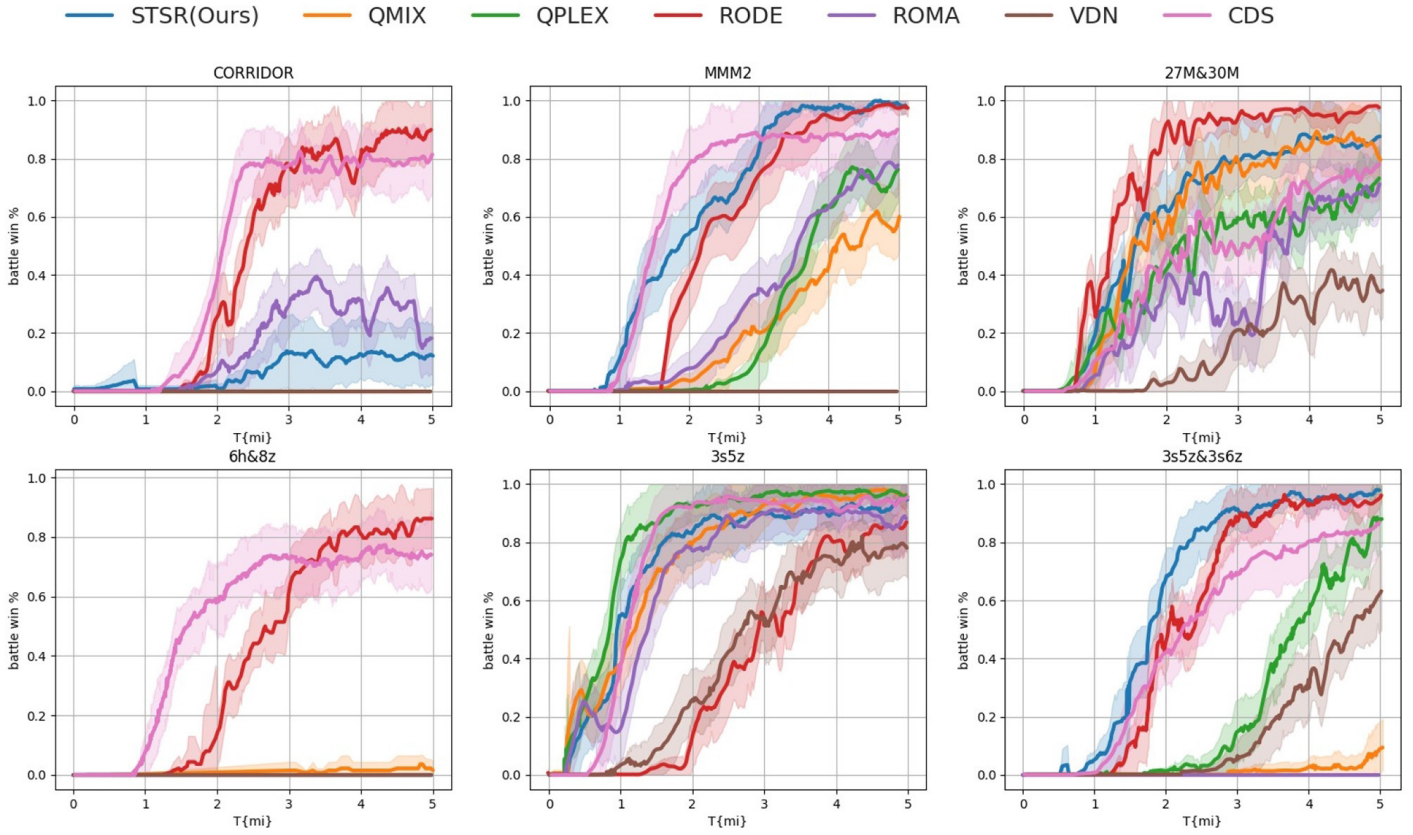
Performance comparison with baselines on all super hard maps and one easy map (3s5z). The baselines compromise VDN (Sunehag et al., 2018), QMIX (Rashid et al., 2020), QPLEX (Wang et al., 2021), role based algorithms ROMA (Wang et al., 2020a), RODE (Wang et al., 2020b), and CDS (Li et al., 2021).

We conducted a comprehensive evaluation of our approach by benchmarking it across all 14 scenarios, categorized in Table 1. Due to space limitations, we present examples of one easy map (3s vs. 5z) and all the super hard maps in Figure 6. Among the tests presented, our proposed method STSR demonstrated the best performance in scenarios 3s5z vs. 3s6z and MMM2, and ranked second in scenarios 3s5z and 27m vs. 30m. These results are not surprising, as our proposal primarily focuses on heterogeneous agents' settings. Compared to role-based methods that cluster agents based on their properties CDS (Li et al., 2021) which seeks to achieve the maximum diversity among individualized behaviors from the shared network., our proposal outperforms in heterogeneous settings, particularly in the speed of convergence. Clustering agents of the same kind and sharing parameters among them is a natural choice. We believe that our proposed agent clustering method is more stable and consistent, enabling more efficient use of generated experience to train policy networks. In contrast, role-based methods may require more interactions with the environment to better understand the agents' properties and assign roles, which may cause a delay in convergence. The size of the agents in these 2 scenarios may be well-suited for our proposed method. In the map 3s5z vs. 3s6z there are 3 Stalkers and 5 Zealots, while in the map MMM2 there is 1 Medivac, 2 Marauders, and 7 Marines. The size of each agent type is not too large or too small, making it appropriate to share the same type of parameters. In contrast, in the map bane vs. bane there are 20 Zerglings and four Banelings. The size of the Zerglings is too large and may require clustering in advance. One surprising outlier is the *easy* scenario 3s5z for which QPLEX exhibits the best performance, surpassing our proposal and the role-based method by a large margin. We hypothesize that this is because these maps do not require significant exploration or distributed policy training. The limited experience can be better utilized by training on a single, fully-parameter-shared network.

**Table 1:** Categories of the SMAC scenarios and their corresponding difficulties, ally units, and agents type.

**Difficulties**	**Name**	**Ally units**	**Agents type**
*Easy*	2*s*3*z*	2 Stalkers & 3 Zealots	Heterogeneous
	3*s*5*z*	3 Stalkers & 5 Zealots	Heterogeneous
	1*c*3*s*5*z*	1 Colossus, 3 Stalkers & 5 Zealots	Heterogeneous
	5m_vs._6m	5 Marines	Homogeneous
	10m_vs._11m	10 Marines	Homogeneous
*Hard*	2s_vs._1sc	2 Stalkers	Homogeneous
	3*s*_*vs*._5*z*	3 Stalkers	Homogeneous
	2c vs. 64zg	2 Colossi	Homogeneous
	Bane vs. bane	20 Zerglings & 4 Banelings	Heterogeneous
*Super hard*	3s5z_vs_3s6z	3 Stalkers & 5 Zealots	Heterogeneous
	6*h*^−^vs. 8*z*	6 Hydralisks	Homogeneous
	27m_*vs*_30m	27 Marines	Homogeneous
	Corridor	6 Zealots	Homogeneous
	MMM2	1 Medivac, 2 Marauders & 7 Marines	Heterogeneous

In contrast to achieving the best performance on heterogeneous agent scenarios, our proposed STSR is less efficient in homogeneous agent settings compared to its counterparts from role-based algorithms such as RODE (Wang et al., 2020b) and ROMA (Wang et al., 2020a), and diversity oriented parameter sharing algorithm CDS (Li et al., 2021). Role-based algorithms employ different principles in clustering small groups of agents automatically and then apply role-based policies to improve the overall performance, whileour approach relies purely on the agents' unit types. CDS (Li et al., 2021) leverages information-theoretical regularization to maximize the mutual information between agents' identities and their trajectories with the purpose to promote learning sharing among agents while keeping necessary diversity. Thus for the scenarios with homogeneous agents which cannot be clustered and achieve sufficient diversity from the environmental exploration and agents behavior the performance of our approach is comparatively lower than the aforementioned counterparts. Empirically, we have observed that the performance on scenarios with homogeneous agents can be enhanced by employing random clustering as an initial step. We plan to conduct a detailed investigation of this phenomenon in our forthcoming research on clustering size, the initial settings, etc.

Our method introduces a hierarchical parameter sharing scheme, wherein parameters are fully shared among agents of the same type and partially shared among agents of different types through hard parameter sharing. By sharing parameters, agents can leverage each other's experiences and exploit common patterns in the environment based on their similarities. This approach simplifies training and enables efficient knowledge transfer. In contrast, role-based MARL assigns specific roles or tasks to individual agents, defining their unique responsibilities and objectives. Each agent possesses its own set of parameters optimized for fulfilling its designated role. Roles can be predefined or learned during training. This approach fosters specialization and coordination among agents, as they concentrate on specific tasks or functions. While role-based MARL excels in handling complex scenarios and adapting to diverse environments, it may necessitate more intricate training algorithms and coordination mechanisms. CDS (Li et al., 2021) propose an information-theoretical regularization to maximize the mutual information between agents' identities and their trajectories, which encourages extensive exploration and diverse individualized behaviors. It introduce agent-specific modules in the shared neural network architecture, which are regularized by L1-norm to promote learning sharing among agents while keeping necessary diversity. Compared to our proposed STSR and role-based methods, CDS (Li et al., 2021) allows for more flexibility in fostering agent specialization and achieving diversity in individualized behaviors. However, without clustering-based group tactics, it results in low efficient utilization of experience.

#### 5.2.2. Ablation study

**Figure 7:**
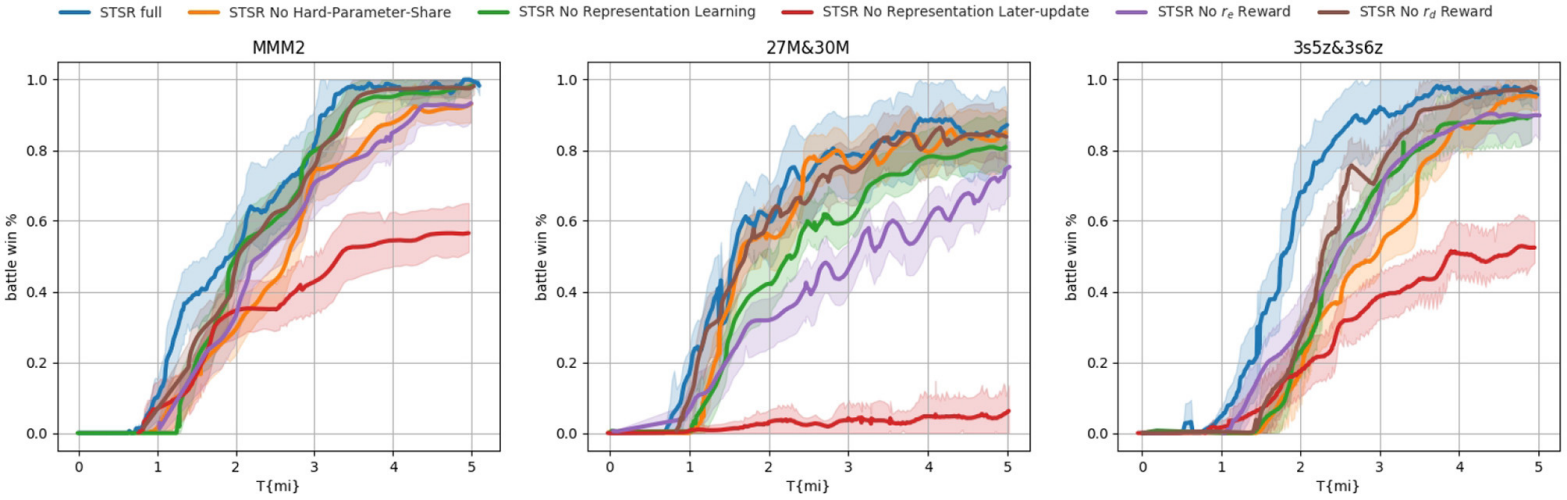
Ablation study on 3 best-performing scenarios. All components make positive contributions to the overall performance.

To better understand the contributions of each component, we conducted an ablation study on three scenarios with the best performance: 27m vs. 30m, 3s5z vs. 3s6z, and MMM2. Among these scenarios, 3s5z vs. 3s6z and MMM2 are heterogeneous, while 27m vs. 30m is homogeneous. The performance of the ablation study can be viewed in Figure 7. According to the results we presented, all components make positive contributions to the overall performance. Among all the curves, *STSR No Representation Later-update* had the worst performance, implying that the original representation from supervised learning is not sufficient for a hard-parameter sharing basis, and a later updated data procedure is necessary. Meanwhile, the curves *STSR No Representation Learning* are not as good as *STSR full* on all scenarios, which means that even if an initial value settled on the hard-parameter sharing basis may not be sufficient, it can still help to quickly approach the proper basis. For the scenario 27m vs. 30m, there is no difference in performance between *STSR full, STSR No Hard-Parameter-Share*, and *STSR No* *r*_*d*_ *Reward*. This result is not surprising since these two components mainly work for heterogeneous agents, and 27m vs. 30m is a homogeneous scenario. The comparison between *STSR No* *r*_*d*_ *Reward* and *STSR full* on the other two scenarios shows that the application of *r*_*d*_ can help to more quickly approach the hard-parameter sharing layer, especially at the beginning. Such a contribution is decreased following the later update of the sharing layer. The reward *r*_*e*_ can help achieve better performance on all scenarios regardless of whether they are homogeneous or heterogeneous (presented on *STSR No* *r*_*e*_ *Reward*), by encouraging environment exploration. The performance of *STSR full* suggests that the utilization of hard-parameter sharing may not approach the capability to largely improve performance, but it does speed up the training process.

In conclusion, the ablation study found that all components make positive contributions to overall performance. The study also showed that even an initial value settled on the hard-parameter sharing basis may not be sufficient, but it can still help to quickly approach the proper basis. The utilization of hard-parameter sharing may not largely improve performance, but it does speed up the training process. The application of *r*_*d*_ can help to more quickly approach the hard-parameter sharing layer, especially at the beginning, and such a contribution decreases following the later update of the sharing layer. The reward *r*_*e*_ can help achieve better performance in all scenarios by encouraging environmental exploration.

### 5.3. Diverged representation embedding training

**Figure 8:**
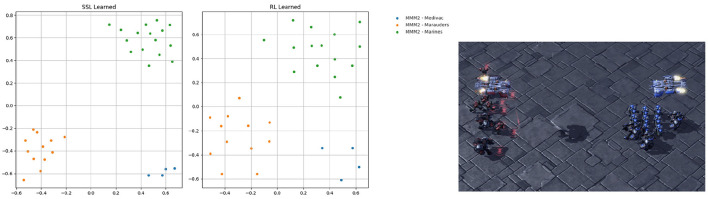
Visualization of representation embeddings of scenario MMM2 projected to 2D space on step 2500, updated, respectively, through ever-existing self-supervised learning and RL. There are 3 kinds of points representing 3 kinds of agents type for scenario MMM2, which are marines, marauders, and medivac.

In our proposal, we introduce a novel approach for representation embedding. Initially generated through self-supervised learning, the representation embedding is duplicated and diverged into two branches. One branch is updated using reinforcement learning to handle the observation for RL, while the other branch is continuously updated to guide the intrinsic reward. Although these two branches serve different purposes, they function similarly to the representation of the common agent's observation and action. To gain a deeper understanding of the functions and capabilities of these two embedding representations, we conducted an experiment comparing their centralization and clustering properties. To achieve this, we projected the embeddings onto a 2D space, as depicted in Figure 8, using the scenario MMM2 as an illustrative example.

In the MMM2 scenario, which comprises a heterogeneous composition of 1 Medivac, 2 Marauders, and 7 Marines facing 1 Medivac, 3 Marauders, and 8 Marines, it is essential to foster effective cooperation among different agent types to fully exploit the advantages of each unit. Our observation revealed that while both forms of embedding clustered within their respective groups, their concentration levels varied. This indicates that both embeddings are capable of effectively distinguishing the observations and actions of different unit types, albeit with varying degrees of concentration, resulting in distinct functional characteristics.

The self-supervised embedding, which is supervised by self-supervised learning, exhibited a higher level of concentration, while the RL-led embedding showed slightly more diversity among individual points. We hypothesize that the self-supervised embedding prioritizes forming distinctive representations for each unit type, reinforced by intrinsic rewards. Hence, the dense concentration in the self-supervised embedding as a result of this objective. On the other hand, the RL embedding focuses on obtaining maximum rewards, the distinctiveness of representation for each individual agent will access a more proper reaction for each agent. Therefore, the RL embedding aims to strike a balance between representing the unit type and the individual agent's characteristics.

#### 5.3.1. Representation transferability and curriculum learning

In this paper, we propose a method that can transfer learned policies to new agents without requiring the entire system to be retrained. This is achieved by duplicating common representations and sharing parameters among agents of the same type. An additional benefit of this approach is that it can be easily applied to tasks involving curriculum learning, where agents of different types are gradually introduced. To accomplish this, we first identify the type of the incoming agents, then average the outputs of agents of the same type from the updated representation. Next, we duplicate the policy parameters of the agent type and apply them to the new agent. By duplicating policies and representations, we ensure that learned policies can be transferred to tasks with varying numbers of agents. This makes our proposed method versatile and applicable to a wide range of tasks without the need for additional training.

**Figure 9:**
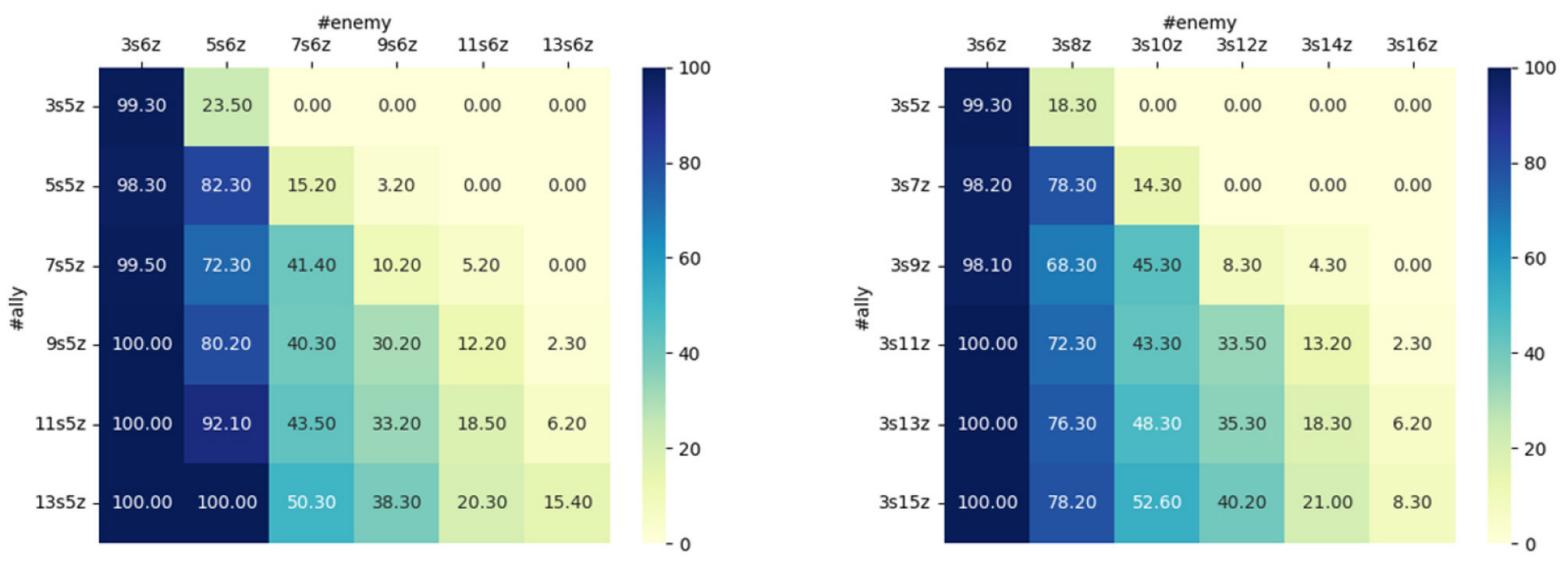
Transferability on the unseen maps on 3s5z vs. 3s6z without further training on the new maps.

We evaluated the transferability of our method on the SMAC benchmark by sorting allies and enemies based on their relative distances to an agent and including information on the nearest ones while keeping the observation length fixed. Figure 9 shows the win rates of the policy learned from the map 3s5z vs. 3s6z on various maps without further policy training. In the original task, 3 Stalkers and 5 Zealots face 3 Stalkers and 6 Zealots. We designed 2 types of maps which, respectively, increased the number of Stalkers and Zealots for both the number of allies and enemies to test the transferability of different agents' types. We observed that the transferability of STSR was evident from the learned policy and still has a good performance on new maps especially when both sides increase their agents' numbers. Additionally, our proposed method is easy to extend for the transferring to the increased size of agents which may help to provide a promising result in curriculum learning.

## 6. Conclusion and future work

Overall, this research provides a fresh perspective on addressing the challenges of parameter sharing in multi-agent reinforcement learning, particularly in heterogeneous environments. The proposed approach not only enables agents to learn from each other but also improves the overall performance of the system. These contributions allow for specialization among heterogeneous agents while still promoting experience sharing, and make it easier to incorporate the hard-parameter-sharing scheme. The proposed method outperforms current mainstream algorithms, particularly for heterogeneous agents, and can be considered a more general and fundamental structure for heterogeneous agent reinforcement learning. Our work is the first to introduce a multi-task network parameter-sharing scheme to MARL and to utilize a supervised learning method for generating a shared input/output representation. Additionally, our proposed intrinsic reward is based on the prediction of supervised learning and its input/output representation, which can stimulate more exploration and enhance the representability of this representation without requiring extra effort. Overall, our contributions provide a promising direction for addressing the challenges in MARL and improving performance for heterogeneous agents.

Based on our experiments, it was observed that one of the bottlenecks in our work is its focus solely on scenarios with heterogeneous agents. It is not well-suited to scenarios with homogeneous agents, and even for heterogeneous scenarios with a large group of the same kind of agents. In comparison with role-based MARL methods, a smaller clustered parameter-sharing group is required. We have empirically noted that a random clustering of homogeneous agents can outperform the baselines and our proposed work. For our future work, we plan to conduct further research to gain a better understanding of the principles behind this observation and make appropriate improvements to the parameter-sharing groups.

## Data availability statement

The raw data supporting the conclusions of this article will be made available by the authors, without undue reservation.

## Author contributions

All authors listed have made a substantial, direct, and intellectual contribution to the work and approved it for publication.
